# The Biofilm Lifestyle Involves an Increase in Bacterial Membrane Saturated Fatty Acids

**DOI:** 10.3389/fmicb.2016.01673

**Published:** 2016-10-28

**Authors:** Florence Dubois-Brissonnet, Elsa Trotier, Romain Briandet

**Affiliations:** Micalis Institute, Institut National de la Recherche Agronomique, AgroParisTech, Université Paris-SaclayJouy-en-Josas, France

**Keywords:** biofilm, membrane lipids, fatty acids, membrane fluidity, lipidomics

## Abstract

Biofilm formation on contact surfaces contributes to persistence of foodborne pathogens all along the food and feed chain. The specific physiological features of bacterial cells embedded in biofilms contribute to their high tolerance to environmental stresses, including the action of antimicrobial compounds. As membrane lipid adaptation is a vital facet of bacterial response when cells are submitted to harsh or unstable conditions, we focused here on membrane fatty acid composition of biofilm cells as compared to their free-growing counterparts. Pathogenic bacteria (*Staphylococcus aureus, Listeria monocytogenes, Pseudomonas aeruginosa, Salmonella* Typhimurium) were cultivated in planktonic or biofilm states and membrane fatty acid analyses were performed on whole cells in both conditions. The percentage of saturated fatty acids increases in biofilm cells in all cases, with a concomitant decrease of branched-chain fatty acids for Gram-positive bacteria, or with a decrease in the sum of other fatty acids for Gram-negative bacteria. We propose that increased membrane saturation in biofilm cells is an adaptive stress response that allows bacteria to limit exchanges, save energy, and survive. Reprogramming of membrane fluidity in biofilm cells might explain specific biofilm behavior including bacterial recalcitrance to biocide action.

## Introduction

Biofilms are surface-associated communities embedded in a self-produced extracellular polymeric substances and organized in a three-dimensional structure (Costerton et al., 1987; Giaouris et al., 2015). Foodborne pathogens such as *Listeria monocytogenes* (Smith et al., 2009; Carpentier and Cerf, 2011; Srey et al., 2013), *Salmonella* (Smith et al., 2009; Dubois-Brissonnet, 2012; Giaouris et al., 2014; Yang et al., 2016), *Yersinia enterocolitica* (Mosteller and Bishop, 1993), *Staphylococcus aureus* (Di Ciccio et al., 2015), *Escherichia coli* EHEC (Smith et al., 2009), or *Campylobacter* (Smith et al., 2009) form biofilms on food contact surfaces. Microbial deposits on wet surfaces, in particular floors and surfaces of equipment, are now recognized to be the main cause of pathogen persistence in food environments. They can be a periodic source of bacterial pathogens contaminating food products during their transformation and can thus lead to foodborne intoxications (Stocki et al., 2007; Dubois-Brissonnet, 2012; Giaouris et al., 2012; Srey et al., 2013). As an example, surface contamination was identified as a cause in 34% of outbreaks occurring in collective catering in France (INVS, 2014).

The essential contributing factor explaining pathogen persistence in food-processing environments is the lack of disinfection efficacy. Despite extensive use of disinfectants on food-contact surfaces, these procedures are less effective when applied on biofilms compared to their effects on free-living bacteria (Bridier et al., 2011). Depending on the species and the biocide considered, biofilms cells can be 1–1000 times more tolerant than their planktonic counterparts. The mechanisms involved in biofilm tolerance to antimicrobial treatments are multifaceted. They are in particular associated with heterogeneous metabolic activity and cell adaptive responses that are specific to physical and chemical microenvironments within the biofilm (e.g., varied conditions of pH, osmotic strength, nutrients or exposure to sublethal concentrations of biocide; Bridier et al., 2011; Giaouris and Nesse, 2015). Biofilm cell transcriptomic or proteomic profiles are studied since 2000’ (Sauer, 2003; Resch et al., 2006; Vilain and Brozel, 2006), revealing up- or down- regulated functions at different stages of biofilm formation compared to their free cell counterparts. Surprisingly, few studies focused on biofilm lipidomics despite the involvement of membrane fatty acid composition in bacterial adaptation to fluctuating environments (Denich et al., 2003). Lipids are the main constituents of the cytoplasmic membrane and are essential for bacterial integrity, survival and growth, by allowing passive permeability of hydrophobic compounds and by modulating the function of membrane-associated proteins. Fluidity and permeability of the membrane rely on lipid acyl chain composition (Parsons and Rock, 2013). To survive despite environmental disturbances such as sub-optimal temperatures or the presence of toxic compounds at sub-lethal doses and to sustain optimum membrane fluidity, bacterial cells can alter the acyl chain structure of membrane glycerophospholipids by changing the ratios of: (1) saturation to unsaturation, (2) *cis* to *trans* unsaturation, (3) branched to non-branched structures, (4) acyl chain length, and (5) the synthesis of cyclopropane fatty acids (CFA; Denich et al., 2003; Loffhagen et al., 2007). It has also been shown that free exogenous fatty acids (EFA) available in the growth environment can alter the bacterial fatty acids (FA) composition (Brinster et al., 2009, 2010).

The objective of this study was to investigate how membrane fatty acid composition is adjusted when pathogenic bacteria are grown in the biofilm state and further, how this adjustment is done when some free exogenous FA are available in the environment. To this end, we first compared FA profiles of two Gram-positive bacteria, *S. aureus* and *L. monocytogenes*, and two Gram-negative bacteria, *Pseudomonas aeruginosa* and *Salmonella* Typhimurium, when grown in the planktonic state (harvested in exponential or stationary phase) or in the biofilm state (on polystyrene plates). Afterward, we investigated the impact of medium supplementation with free saturated or unsaturated exogenous FA on *S. aureus* profiles in both planktonic or biofilm states.

## Materials and Methods

### Strain and Growth Conditions

*Staphylococcus aureus* RN 4220, *S. aureus* HG003, *L. monocytogenes* Scott A, *S.* Typhimurium ATCC 13311, and *P. aeruginosa* ATCC 11442 were used in this study. They were inoculated in Tryptone Soya Broth (TSB; Biomérieux, Marcy l’Etoile, France) or in Brain Heart Infusion (BHI; Oxoid, Basingstoke, UK) at 1% v/v with a standardized inoculum (~10^8^ cells/mL) obtained after two subcultures in the same broth. Cultures were incubated and harvested in exponential phase or stationary phase for fatty acid analysis.

When indicated, an exogenous fatty acid (EFA) is added to the culture medium: myristic acid (C14) or palmitic acid (C16) [saturated fatty acids (SFA)], oleic acid (C18:1cis9; monounsaturated fatty acid), linoleic acid (C18:2w6), linolenic acid (C18:3w6), or arachidonic acid (C20:4w6) [polyunsaturated fatty acids]. EFA were first dispersed in a bovine serum albumin (BSA) solution before addition to the growth medium. Final EFA concentration in BHI was 0.9 mM.

### Biofilm Formation

Biofilms were grown on the polystyrene base of 96-well polystyrene microtiter plates (Greiner Bio-One 655090, France) as previously described (Bridier et al., 2010). 250 μL of bacterial subculture (~10^6^ cells/mL) were poured into the wells and adhesion was done by sedimentation for 2 h. Subsequently, the planktonic bacterial suspension was removed and 250 μL of medium were added in each well. Microtiter plates were incubated for 48 h without shaking to allow biofilm development.

### Structural Biofilm Properties Observed by Confocal Laser Scanning Microscopy (CLSM)

Following incubation, biofilms were rinsed with 150 mM NaCl and refilled with TSB or BHI containing 5 μM Syto 9 (1:1000 dilution from a Syto 9 stock solution at 5 mM in DMSO; Invitrogen, France), a cell-permeable green fluorescent nucleic acid marker. The plate was then incubated in the dark at 30°C for 20 min to enable fluorescent labeling. Images were acquired using a Leica SP2 AOBS confocal laser scanning microscope (Leica Microsystems, France) at the MIMA2 microscopy platform^1^. The excitation laser wavelength used for Syto 9 was 488 nm, and emitted fluorescence was recorded within the range 500–600 nm. Images (512 × 512 pixels) were acquired through a 63 × Leica oil immersion objective (numerical aperture, 1.4) with a z step of 1 μm and a frequency of 400 Hz. 3D projections were generated with the Easy 3D IMARIS function (Bitplane, Zurich, Switzerland). Biofilms structural parameters [biovolume (μm^3^), mean thickness (μm), and roughness (μm)] were extracted from image series using the PHLIP Matlab routine^2^. Each value presented is the average of six image series acquired in three independent experiments.

### Membrane Fatty Acid Analysis

Bacterial planktonic cultures grown as described above were harvested by centrifugation (7000 *g*, 20°C, 10 min) in exponential or stationary phase according to OD growth curves. Pellets were washed twice with 0.1% triton X-100 in order to remove unincorporated EFA when present. Biofilm cells for each species were collected from ten 96-plate wells. Biofilms were first rinsed once in 0.1% triton X-100 before biofilm cells were removed from polystyrene wells by scratching and re-suspended and washed again in 0.1% triton X-100. Extraction and methylation of fatty acids were carried out directly on bacterial pellets. Fatty acids of whole cells were first saponified and esterified by methanolic NaOH and methanolic HCl (first step: 1 mL NaOH 3.75 mol/l in 50% v/v methanol solution for 30 min at 100°C; second step: addition of 2 mL HCl 3.25 mol/l in 45% v/v methanol solution for 10 min at 80°C; Méchin et al., 1999). Fatty acid methyl esters were extracted with a diethyl ether/cyclohexane solution (1:1 v/v). The organic phase was at the end washed with a dilute base (NaOH 0.3 mol/l). Analytical gas chromatography of fatty acid methyl esters was carried out on a 6890HP system (Agilent Technologies, Santa Clara, CA, USA) equipped with a DB5 capillary column (Agilent Technologies, Santa Clara, CA, USA) and a flame-ionization detector. Column temperature was set at 150°C for 4 min and then increased to 250°C at the rate of 4°C/min. Data were acquired using a HPCORE ChemStation system (Agilent Technologies, Santa Clara, CA, USA) and expressed as a percentage of the total area. Fatty acids were identified using fatty acid methyl ester standards and grouped in classes: saturated fatty acids (SFA), unsaturated fatty acids (UFA), hydroxylated fatty acids (HFA), cyclopropane fatty acids (CFA), branched-chain fatty acids (BCFA). Results are the average of at least eight profiles (two injections of four to nine extractions from independent cultures) for each condition.

### Statistics

ANOVA variance analyses were performed using Statgraphics software (Manugistic^TM^, Rockville, MD, USA). Evaluated factors were considered as statistically significant when *P*-values associated with the Fischer test were below 0.05.

## Results

### Biofilm Cells Are Rich in Saturated Fatty Acids

Fatty acid profiles of *S. aureus* HG003, *L. monocytogenes* Scott A, *S.* Typhimurium ATCC 13311 and *P. aeruginosa* ATCC 11442 were compared when grown in TSB at 30°C in planktonic state (exponential or stationary phases) or biofilm state. The fatty acid profiles of stationary phase cultures grown in planktonic conditions are shown as reference in Supplementary Table S1.

**FIGURE 1:**
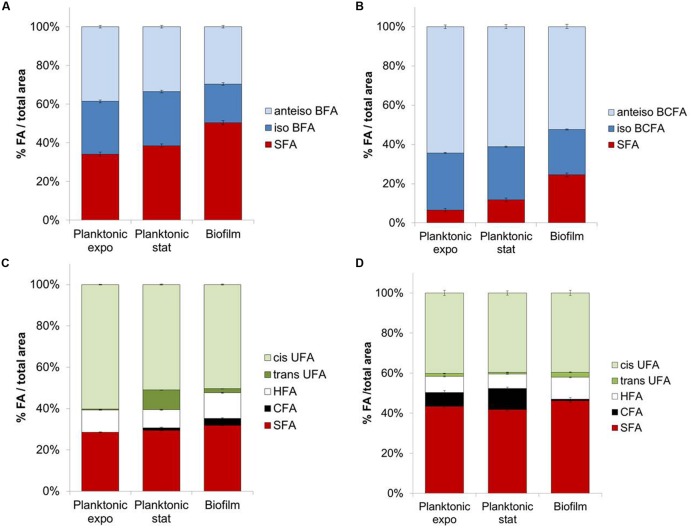
**Fatty acids composition for planktonic (exponential and stationary phases) or biofilm states of (A) *Staphylococcus aureus* HG003, (B) *Listeria monocytogenes*, (C) *Pseudomonas aeruginosa*, (D) *Salmonella* Typhimurium**.

In planktonic cultures, SFA content significantly increased (*P* < 0.05) between exponential and stationary phases for *S. aureus* (+4.3%; **Figure 1A**), *L. monocytogenes* (+5.3%; **Figure 1B**), and *P. aeruginosa* (**Figure 1C**). Concomitant decreases are seen in amounts of *anteiso*-BCFA for both Gram-positive bacteria, and in *cis*-UFA at the expense of *trans*-UFA for *P. aeruginosa* (*P* < 0.05). Interestingly, decreases in *anteiso*-BCFA and UFA may lead to decreased membrane fluidity in stationary phase cells.

Biofilm cells exhibit different FA profiles compared to planktonic cells in both exponential and stationary phases. SFA content in biofilm cells is significantly higher than in planktonic cells for the four tested bacteria (*P* < 0.05) (**Figures 1A–D**). This increase is considerable for both Gram-positive bacteria, with, respectively, +12 and +12.7% compared to stationary phase for *S. aureus* and *L. monocytogenes*. This effect is mainly due to the increase of C16 content in *S. aureus* (+11.7%) and of C16 and C18 contents for *L. monocytogenes* (+4.4 and 6.4%, respectively). Both *iso*-C15 and *anteiso*-C15 BCFA contents significantly decrease in biofilm cells (*P* < 0.05) compared to their planktonic counterparts in stationary phase (respectively, -7.1 or -5.5% *iso*-C15 and -8.5 or -13.1% *anteiso*-C15 for *S. aureus* or *L. monocytogenes*). As before the observed increases in SFA and/or decreases in *anteiso*-BCFA species might indicate a more rigid membrane in biofilm than planktonic cells.

**FIGURE 2:**
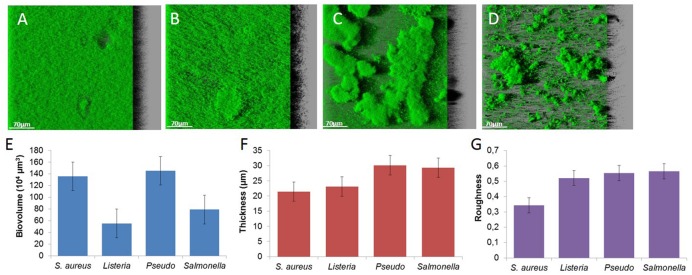
**Images of (A) *S. aureus* HG003, (B) *L. monocytogenes*, (C) *P. aeruginosa*, (D) *S.* Typhimurium biofilms and structural parameters, (E) biovolume, (F) thickness, and (G) roughness**.

Biofilms observed by CLSM exhibit a variety of spatial organization according to the species. *S. aureus* forms flat and dense biofilms characterized by a high biovolume, low thickness and roughness (**Figures 2A,E–G**). *L. monocytogenes* also forms flat structures (lower biovolume, higher thickness and roughness; **Figures 2B,E–G**). The two Gram-negative bacteria trigger typical mushroom-like structures with high thickness and roughness. *P. aeruginosa* produces sparse but larger mushrooms (**Figure 2C**), in contrast to *S.* Typhimurium that produces numerous smaller clusters (**Figure 2D**).

### The Biofilm Lifestyle Alters *S. aureus* Selectivity of Exogenous FA Incorporation

**FIGURE 3:**
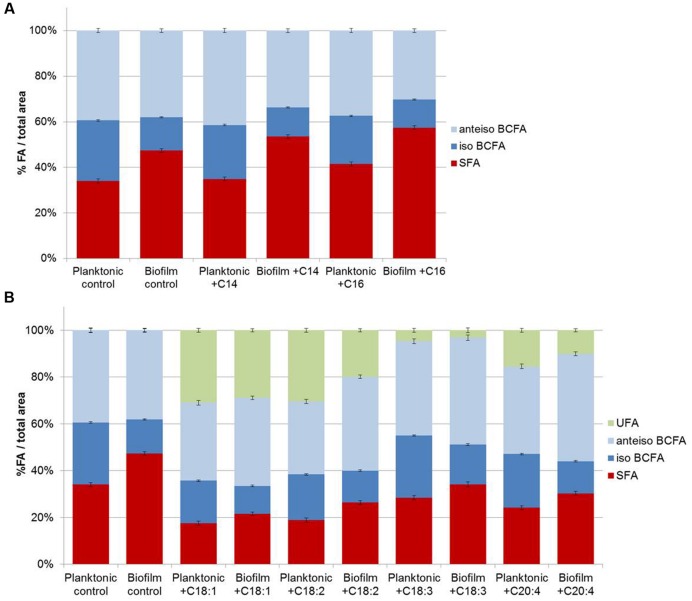
***Staphylococcus aureus* RN 4220 FA composition cultivated in planktonic or biofilm states with or with saturated fatty acids (SFA) supplementation (A) and with or without unsaturated fatty acids (UFA) supplementation (B)**.

Fatty acid profiles of *S. aureus* RN 4220 were analyzed when grown in BHI at 37°C in the planktonic state (stationary phase) *versus* biofilm state in the absence or presence of exogenous FA (saturated or unsaturated FA dispersed in BSA; **Figures 3A,B**). In control conditions (without supplementation), the *S. aureus* RN 4220 qualitative fatty acid profile was similar to that of HG003 strain (Supplementary Table S1). Moreover, cells grown as biofilms also display a significantly higher SFA content (+13.3%) compared to cells in the planktonic state, due to the high increases of C18 and C20 SFA (respectively, +5.5 and +8.3%). At the same time, we detected a high decrease of C15-BCFA (-13.4%) together with a slight increase of *anteiso*- C17 (+2.1%).

Supplementation of saturated or unsaturated FA dispersed in BSA does not modify the *S. aureus* growth curve (data not shown). When exogenous C14 or C16 SFA are available in the culture medium, both planktonic and biofilm *S. aureus* membranes contain more of the added FA (C14 or C16) and more C20 FA. But, as in control conditions, biofilms cells contain more SFA (+18.5 and +15.9% with exogenous C14 and C16, respectively), namely C18 and C20 SFA, and less C15 BCFA than planktonic cells (**Figure 3A**). UFA addition also significantly affected fatty acid profiles of both cell populations and among UFA, C18:1 and C18:2 were more incorporated than C18:3 or C20:4. In contrast to SFA, UFA are less incorporate in biofilm cells as compared to planktonic cells (-2.1, -10.5, -1.5, -5.4% with exogenous C18:1, C18:2, C18:3, C20:4, respectively; **Figure 3B**).

Overall, SFA are significantly higher and *iso*-BCFA are significantly lower in biofilm cells than in planktonic ones (*P* < 0.05). No significant difference is observed for *anteiso*-BCFA. When present, UFA are significantly less incorporated in biofilm cells (*P* < 0.05).

**FIGURE 4:**
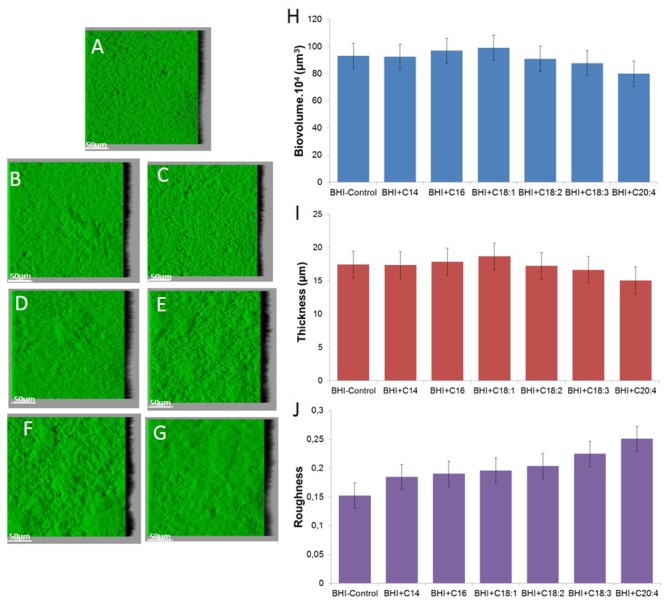
**Images of *S. aureus* RN 4220 biofilms grown without supplementation (A) or with supplementation of fatty acid coupled with bovine serum albumin (BSA) namely C14 SFA (B), C16 SFA (C), C18:1 UFA (D), C18:2 UFA (E), C18:3 UFA (F) and C20:4 UFA (G) and biofilm parameters (H) biovolume, (I) thickness and (J) roughness**.

*Staphylococcus aureus* RN 4220 forms flat biofilm structures, as does strain HG003 (**Figure 4**). Supplementation with SFA or UFA does not significantly impact biofilm biovolume and thickness after 48 h (*P* > 0.05). An increase of roughness can only be observed with C18:3 and C20:4 supplementation in comparison of control (*P* < 0.05).

## Discussion

Numerous studies invoke physiologic state of biofilm cells to explain their increased tolerance to antibiotics and disinfectants compared to their free counterparts. Although membrane is the first integrity barrier of the cell, few studies focused on phospholipidic membrane adaptation when bacteria are grown in biofilm state. In this study, we investigated the impact of the biofilm lifestyle on bacterial fatty acid composition and have shown that FA profiles of biofilm cells differed significantly from those of planktonic cells for four different bacterial species that show various spatial structuration of the biofilm (flat or mushroom-like structures). In all cases, FA profiles of biofilm cells contain significantly higher proportions of SFA compared to planktonic ones. In Gram-positive bacteria, increases in SFA (in particular long chain FA) are concomitant to decreases in BCFA content. This FA shift in biofilm cells was previously described in *L. monocytogenes* on simply adhered cells (Gianotti et al., 2008). It was also described that *Rhodococcus erythropolis* produced in general 5% more SFA than their planktonic counterparts in Muller-Hinton broth, even if lipid composition mainly depends on the type of surface and medium composition (Rodrigues and de Carvalho, 2015). The increase of SFA leads to a higher phase transition temperature, density of packing, and bilayer stability (Denich et al., 2003). Moreover, increases in long chain SFA may increase penetration of FA into the bilayer, favor interactions between acyl chains, and increase bilayer rigidity (Denich et al., 2003). Bacteria living within a biofilm consortium are surrounded by a polymeric matrix that triggers a heterogeneous environment where nutrients and oxygen are less available than in a liquid medium (Bridier et al., 2011). Nutrient stress was previously shown to increase SFA content in planktonic cells (Guckert et al., 1986). But high SFA levels in biofilm cells could also reflect a specific physiological state induced during the early phases of attachment, as shown for *L. monocytogenes* (Gianotti et al., 2008).

Besides well-known bacterial ability to adapt FA synthesis under environmental conditions, it was demonstrated that bacteria can incorporate free exogenous FA from its environment to its membrane. Incorporation of serum FA into membranes allows *S. aureus* survival and growth in the presence of antibiotics targeting the FA synthesis pathway (Brinster et al., 2010). *Vibrio cholerae* was also shown to incorporate long chain PUFA present in bile into its membrane phospholipids (Giles et al., 2011). In this context, we also investigated the impact of free exogenous FA on FA membrane profiles of biofilm *S. aureus* cells in comparison to planktonic counterparts to evaluate how ubiquitous is the phenomenon of membrane saturation in biofilm cells. Planktonic cells and biofilm cells can both incorporate FA. Supplementation with SFA, namely C14 or C16, or with UFA, namely C18:1 or C18:2, leads to FA elongation with, respectively, a high increase in C20 for SFA and in C20:1 or C20:2 for UFA. It was recently shown that *S. aureus* can incorporate exogenous FA *via* a fatty acid kinase-dependent pathway (Parsons et al., 2014). SFA and UFA diffuse passively through the cytoplasmic membrane and bind, respectively, to FakB1 and FakB2 fatty acid binding proteins before they are converted to acyl carrier protein. In biofilm cells, as in planktonic cells, SFA and UFA can be incorporated and elongated. FA profiles of biofilm cells always showed higher SFA content compared to their planktonic counterparts (**Figure 3**). Biofilm cells thus display a specific physiological behavior which tends to decrease membrane fluidity, leading to fewer exchanges between bacteria and their environment, and likely improving survival in a harsh environment (MacGarrity and Armstrong, 1975).

For Gram-negative bacteria, such as *P. aeruginosa* or *S.* Typhimurium, the increase of SFA content in biofilm cells compared to planktonic ones is lower than for Gram-positive bacteria. Membrane composition of Gram-negative bacteria is more complex, containing CFA and *cis-* and *trans*-UFA and membrane fluidity also depends on ratios of *cis-* to *trans*-UFA and of UFA to CFA. In the literature, results about *P. aeruginosa* are contradictory as some authors demonstrated a decrease of membrane fluidity in biofilm cells due to increased long chain FA and decreased BCFA (Benamara et al., 2011), whereas others showed a less rigid membrane by decrease in linear SFA content and decrease in fatty acid length chains (Chao et al., 2010).

## Conclusion

This study demonstrates that the FA content of biofilm cells can be statistically differentiated from those of free cells (exponential or stationary phase) and that SFA content in biofilm cells is always higher than in planktonic cells in similar conditions. But this global approach obviously does not evaluate the level of physiological heterogeneity within the population. Physiological diversity in biofilms has been shown to promote adaptation to stressful conditions and thus to enhance bacterial survival and resistance to antimicrobial agents (Boles et al., 2004). An interesting way to evaluate heterogeneity of FA modifications within the biofilm will be to use fluorescent reporter tools for monitoring gene expression and membrane status on the single cell level.

## Author Contributions

FD-B provided the general concept, designed and supervised the experiments, and wrote the manuscript. ET carried out most experiments. RB participated in designing the study. All authors contributed to the discussion of the research and approved the final manuscript.

## Conflict of Interest Statement

The authors declare that the research was conducted in the absence of any commercial or financial relationships that could be construed as a potential conflict of interest.

## Footnotes

1 http://www6.jouy.inra.fr/mima2

2 https://sourceforge.net/projects/phlip/
