# Supplementary material for: The Biofilm Lifestyle Involves an Increase in Bacterial Membrane Saturated Fatty Acids
Source: Front Microbiol. 2016 Oct 28;7:1673. doi: 10.3389/fmicb.2016.01673 (PMC5083788; doi:10.3389/fmicb.2016.01673)
Supplement: Supplementary file 1 [file Table_1.docx]

SUPPLEMENTARY Table 1: FA composition of the four species in planktonic stationary phase

| Gram-positive bacteria | | | Gram-negative bacteria | | |
| --- | --- | --- | --- | --- | --- |
| FA composition in stationary phase | *S. aureus* HG003 | *L. monocytogenes* | FA composition in stationary phase | *P. aeruginosa* | *S. Typhimurium* |
| *iso*C14 | 3,63±0.82 | 1,97±0.23 | C12 | 2.56±0.2 | 3.7±0.2 |
| C14 | 1,85±0.67 | 3,14±0.45 | C14 | 0.75±0.1 | 5.49±0.30 |
| *iso*C15 | 18,71±2.08 | 16,47±0.79 | C10bOH | 2.04±0.95 | - |
| *anteiso*C15 | 30,28±1.66 | 43,82±1.21 | C12aOH | 4.19±0.16 | - |
| C15 | 0.37±0.3 | 1.46±0.08 | C16 | 25.56±0.6 | 31.91±1.30 |
| *iso*C16 | 1,36±0.41 | 4,36±0.10 | *trans9* C16 | 7.27±0.52 | 0.36±0.47 |
| *anteiso*C16 | - | 0,09±0.14 | *cis9* C16 :1 | 10.09±0.77 | 14.41±1.22 |
| C16 | 7,96±0.64 | 4,61±0.77 | C12bOH | 2.48±0.26 | - |
| *iso*C17 | 2,95±0.25 | 4,22±1.08 | C17 | - | 0.2±0.2 |
| *anteiso*C17 | 2.59±0.33 | 17.26±2.49 | cycloC17 | 0.63±0.13 | 8.14±3.35 |
| C17 | 1,38±0.12 | 0,16±0.28 | C18 | 0.53±0.09 | 0.22±0.2 |
| *iso*C18 | 0,25±0.14 | - | *trans11* C18 :1 | 2.27±0.19 | 0.4±0.25 |
| *anteiso*C18 | - | - | *cis11* C18 :1 | 40.82±0.78 | 25.25±2.66 |
| C18 | 15,99±2.33 | 2,44±1.87 | C14bOH | - | 7.27±1.0 |
| *iso*C19 | 0,63±0.09 | - | cyclo C19 | 0.69±0.08 | 2.36±1.0 |
| *anteiso*C19 | 0,28±0.1 | - |  |  |  |
| C19 | 2,15±0.23 | - |  |  |  |
| *iso*C20 | 0,44±0.34 | - |  |  |  |
| *anteiso*C20 | 0,45±0.31 | - |  |  |  |
| C20 | 8,68±2.22 | - |  |  |  |
